# Effects of structured involvement of the primary care team versus standard care after a cancer diagnosis on patient satisfaction and healthcare use: the GRIP randomised controlled trial

**DOI:** 10.1186/s12875-022-01746-3

**Published:** 2022-06-04

**Authors:** I. A. A. Perfors, C. W. Helsper, E. A. Noteboom, E. A. Visserman, E. B. L. van Dorst, T. van Dalen, M. A. M. T. Verhagen, A. J. Witkamp, R. Koelemij, A. E. Flinterman, K. A. B. M. Pruissen-Peeters, F. M. N. H. Schramel, M. T. M. van Rens, M. F. Ernst, L. M. G. Moons, E. van der Wall, N. J. de Wit, A. M. May

**Affiliations:** 1 Julius Center for Health Sciences and Primary Care, University Medical Center Utrecht, Utrecht University, Utrecht, The Netherlands; 2 Dutch Federation of Cancer Patient Organizations, Utrecht, The Netherlands; 3 Gynaecologic Oncology dept., University Medical Center Utrecht, Utrecht University, Utrecht, The Netherlands; 4 Surgery dept., Diakonessenhuis Utrecht, Utrecht, The Netherlands; 5 Gastroenterology dept., Diakonessenhuis, Utrecht, The Netherlands; 6 Surgery dept., University Medical Center Utrecht, Utrecht University, Utrecht, The Netherlands; 7 Surgery dept., St. Antonius Hospital, Nieuwegein, The Netherlands; 8 Dermatology dept., Diakonessenhuis, Utrecht, the Netherlands; 9 Dermatology dept., St. Antonius Hospital, Nieuwegein, The Netherlands; 10 Department for Lung Diseases and Treatment, St. Antonius Hospital, Nieuwegein, The Netherlands; 11 Pulmonology dept., Diakonessenhuis, Utrecht, The Netherlands; 12 Surgery dept., Alexander Monro Clinics, Bilthoven, The Netherlands; 13 Gastroenterology dept., University Medical Center Utrecht, Utrecht University, Utrecht, The Netherlands; 14 Department for Internal Medicine and Oncology, University Medical Center Utrecht, Utrecht University, Utrecht, The Netherlands

**Keywords:** Cancer, General practitioners, Oncology, Primary care, Patient satisfaction

## Abstract

**Background:**

The growing number of cancer survivors and treatment possibilities call for more personalised and integrated cancer care. Primary care seems well positioned to support this. We aimed to assess the effects of structured follow-up of a primary care team after a cancer diagnosis.

**Methods:**

We performed a multicentre randomised controlled trial enrolling patients curatively treated for breast, lung, colorectal, gynaecologic cancer or melanoma. In addition to usual cancer care in the control group, patients randomized to intervention were offered a “Time Out consultation” (TOC) with the general practitioner (GP) after diagnosis, and subsequent follow-up during and after treatment by a home care oncology nurse (HON). Primary outcomes were patient satisfaction with care (questionnaire: EORTC-INPATSAT-32) and healthcare utilisation. Intention-to-treat linear mixed regression analyses were used for satisfaction with care and other continuous outcome variables. The difference in healthcare utilisation for categorical data was calculated with a Pearson Chi-Square or a Fisher exact test and count data (none versus any) with a log-binomial regression.

**Results:**

We included 154 patients (control *n* = 77, intervention *n* = 77) who were mostly female (75%), mainly diagnosed with breast cancer (51%), and had a mean age of 61 (SD ± 11.9) years. 81% of the intervention patients had a TOC and 68% had HON contact. Satisfaction with care was high (8 out of 10) in both study groups. At 3 months after treatment, GP satisfaction was significantly lower in the intervention group on 3 of 6 subscales, i.e., quality (− 14.2 (95%CI -27.0;-1.3)), availability (− 15,9 (− 29.1;-2.6)) and information provision (− 15.2 (− 29.1;-1.4)). Patients in the intervention group visited the GP practice and the emergency department more often ((RR 1.3 (1.0;1.7) and 1.70 (1.0;2.8)), respectively).

**Conclusions:**

In conclusion, the GRIP intervention, which was designed to involve the primary care team during and after cancer treatment, increased the number of primary healthcare contacts. However, it did not improve patient satisfaction with care and it increased emergency department visits. As the high uptake of the intervention suggests a need of patients, future research should focus on optimizing the design and implementation of the intervention.

**Trial registration:**

GRIP is retrospectively (21/06/2016) registered in the ‘Netherlands Trial Register’ (NTR5909).

**Supplementary Information:**

The online version contains supplementary material available at 10.1186/s12875-022-01746-3.

## Introduction

As cancer incidence is increasing [1] and prognosis is improving [2] more patients live longer with cancer and experience more late effects of treatment [3], in the presence of co-existing chronic conditions [4]. Consequently, the nature of cancer treatment is shifting towards chronic disease management. This change requires more personalised and integrated care, based on individual preferences and medical profile [5, 6]. In primary care based health care systems, general practitioners (GPs) may be best positioned to provide continuous, personalised and integrated care during the cancer care continuum [4, 5, 7].

Traditionally, management of cancer is delivered by in-hospital specialists. Even though patients increasingly want their GP to be involved in their cancer care [8], attempts to structurally involve primary care during cancer treatment so far have not been successful [9].

Aiming to structurally involve primary care in cancer care, we designed an intervention called ‘GRIP’, in close cooperation with medical professionals and patient organisations. The GRIP intervention consists a “Time Out consultation” (TOC) with the GP aimed to initiate primary care involvement during cancer treatment, and subsequent structured follow-up during and after cancer treatment by a home care oncology nurse (HON) in cooperation with the GP.

Earlier we reported the effect of the TOC on perceived shared decision making (SDM) in cancer treatment [10]. We concluded that timely implementation of a TOC in the current cancer care pathway is challenging, mainly because of the tight time schedule between diagnosis and therapy decision. Here we report the effects of the full GRIP intervention in the year after cancer diagnosis on patient satisfaction, healthcare utilisation, quality of life, mental health and self-efficacy (component of patient empowerment), for patients treated with curative intent.

## Methods

### Design

The GRIP study is a multicentre randomised controlled trial (RCT), of which the protocol has been published previously [11]. The intervention was co-designed by representatives of the Dutch Federation of Cancer Patients Organisations, GP’s, HONs, oncologist, hospital nurses and the research team.

The Medical Ethical Committee of the University Medical Center Utrecht concluded that the study did not meet the criteria for full ethical review, since our study did not meet the second mandatory criterion: ‘*Individuals are subjected to actions or have rules of conduct imposed on them’.* (METC protocol nr 15-075C). Informed consent was obtained from all subjects and/or their legal guardian(s). All methods were performed in accordance with the relevant guidelines and regulations.

### Study population

Patients were recruited in four Dutch hospitals, between April 2015 and May 2017 and followed until April 2018. Patients were eligible if aged ≥18 years, newly diagnosed with either breast, colorectal, gynaecological, lung cancer or melanoma, and scheduled for treatment with curative intent.

Patients were excluded in case they were unable to fill in questionnaires, had a major psychiatric disease or personality disorder, already started cancer treatment or if the patient’s GP worked outside the study area or did not agree to participate.

### Sample size

A medium effect size (0.5) was assumed to be a clinically relevant difference in patients’ satisfaction between the two study groups. Using β = 0.8 and α < 0.05, at least 64 patients per study group were required. Accounting for an estimated dropout of 15%, 75 participants in each group were needed.

### Recruitment and randomization

After being diagnosed, patients were recruited in hospital by the treating specialist or oncology nurse. After consent, the researcher contacted the patient by phone the following day to evaluate eligibility and to provide detailed study information. After sending in the signed informed consent forms, patients were randomised to the usual care or intervention group (1:1). For randomisation the researcher used an online randomisation module provided by an independent data centre. Minimisation was applied to ensure balance between the two groups regarding treating hospital and cancer type. Blinding was not possible due to the nature of the intervention.

### Usual care

Usual care in the hospital is to a generally protocolled and differs depending on cancer type, hospital, patient and caretaker characteristics and patients’ preferences. Treatment options are discussed in a multidisciplinary team and generally follow national guidelines. Cancer care in the hospital is usually delivered by medical doctors specialised in oncology and oncology nurses.

Primary care is not involved in cancer treatment on a structural basis. The GP receives information on the diagnosis and treatment plan by phone or by mail. Hereafter, some GPs may contact the patient, but in most cases they participate during cancer treatment only on patient’s request. Supporting primary care services are only involved if considered necessary. HONs are often involved in a palliative setting, but only incidentally during curative treatment.

The timing of questionnaires depended on the duration of primary cancer treatment. If primary treatment lasted more than 9 months, all questionnaires were provided. If primary treatment was completed between 6 and 9 months after inclusion, T5 was provided 3 months after the end of primary treatment. In this case, the T4 questionnaire was omitted. If primary treatment was completed within 6 months after inclusion, T3 was planned at completion of treatment and T5 3 months later. The remaining questionnaires were omitted. Consequently, every patient received at least the questionnaires from T0, T1, T3 and T5.

### Intervention

**Fig. 1:**
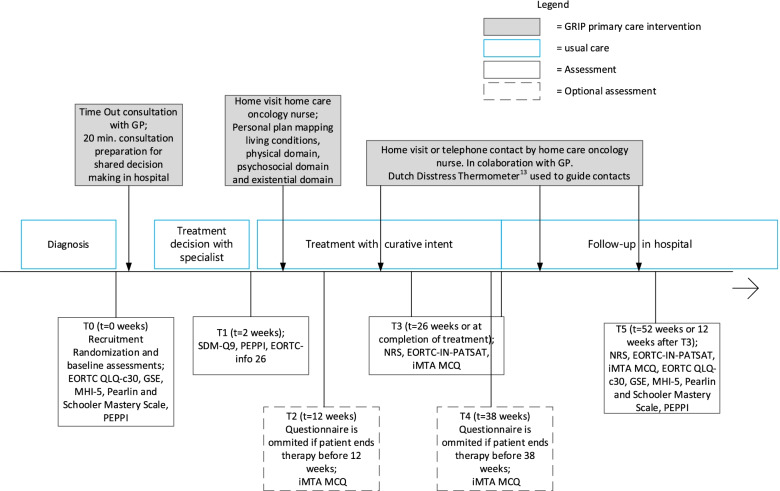
GRIP study, with the usual care, intervention and assessments displayed in time (not on scale). Abbreviation: GP; General practitioner

In addition to usual care, patients in the intervention group were offered structured guidance from primary care. The intervention consisted of two components, TOC and involvement of HONs as displayed in Fig. 1.

#### Time out consultation (TOC)

Intervention patients were advised to make a TOC appointment with their GP. The TOC is a 20-minute consultation, which should be planned between diagnosis and treatment decision in the hospital. The TOC aims to initiate primary care involvement after diagnosis and prepare patients for SDM in the hospital. For this consultation, the GP was instructed to give psychosocial guidance, create awareness that a choice of treatment exists and to instruct the patient to use the three questions model (Shepherd et al. 2011) during the final discussion on the treatment options in the hospital [12]. The three questions are: What are my options? What are the possible benefits and harms of those options? How likely are the benefits and harms of each option to occur for me? This model has demonstrated to improve the quality of information about therapeutic options and to stimulate patients’ involvement in the treatment decision [12].

#### Follow-up care from primary care

During the TOC, joint guidance by the GP and a HON was offered to the patient. HONs are part of primary care and are used to providing information, organise care and give psychological care to patients with cancer and their families; mostly in palliative setting and for the current study this was extended to the curative setting. If a patient accepted HON guidance, the HON was notified and contacted the patient to plan a visit at the patient’s home. During this visit, the HON explained his/her role and made a personal support plan together with the patient. In this plan, the patient’s situation was mapped on four domains: living conditions, physical domain, psychosocial domain and existential domain. If one of the domains required active support the HON discussed the required actions with the patient and if necessary with the GP.

The number, type, and duration of HON contacts was patient-driven, but at least 3 contact moments were recommended; including one home visit during active cancer treatment and two follow up contacts in the 3 months after completion of active cancer treatment. To guide the content of all contact moments the Dutch Distress Thermometer [13] was used. This instrument includes items on five domains (i.e., practical, social, emotional, spiritual, physical), for which patients are asked to rank their level of distress [13]. The HON reported the condition of the patient and required actions to the GP. The hospital was also informed by the HON, in case supportive care was started based on the HON’s consultations (e.g., consultation of a psychologist, physiotherapist or dietician) or when treatment-specific questions arose.

### Intervention training

All the participating HONs were nurses with a specialised training in oncology and had more than 2 years of clinical experience. In addition, they received a 4-hour training provided by the GRIP study team. GPs received basic information on the GRIP study by their GP cooperative organisations at the start of the study. The GPs of intervention patients received the necessary training to perform a TOC and the subsequent intervention individually by phone, and also via email and online.

### Outcomes

Primary outcomes were patient satisfaction with care and healthcare utilisation during the year after inclusion. Secondary outcomes were health related quality of life (hrQoL), mental health and self-efficacy.

### Data collection

Patient reported outcomes and use of paramedical care were collected using questionnaires, which were assessed at baseline (T0), after 2 weeks (T1), every 3 months (T2, T3, T4) and up to 12 months after inclusion (T5). Some questionnaires were omitted if the therapy was shorter than 9 months. Details are depicted in Fig. 1. We assessed the timing of questionnaire assessment of T3 and T5 within the cancer care pathway.

Questionnaires were filled in online or on paper. Non-responders received two reminders by e-mail after two and 5 days and were contacted by phone by the researcher if non-response persisted.

Healthcare utilisation was retrieved from the Electronic Medical Records (EMR) registrations in primary care and hospital. These EMR data include free text and coded data describing daily care, i.e., consultation and referral descriptions, medication and diagnostic information.

GP characteristics and rurality were collected from public Dutch online databases for GP experience [14, 15]. From the hospital EMR, we extracted comorbidities, date of diagnosis, cancer stage, date of treatment decision and completion of active treatment. We defined the date of completion of active treatment as the date of first follow-up contact with their treating physician.

### Questionnaires

Patient satisfaction with care was measured with the European Organisation for Research and Treatment of Cancer Satisfaction with care questionnaire (EORTC-INPATSAT-32) [16] and with a Numeric Rating Scale (NRS). EORTC-INPATSAT-32 is a validated questionnaire and consists of 32 questions measuring patients’ appraisal of hospital doctors and nurses, as well as aspects of care organisation and services [16]. We adjusted the EORTC-INPATSAT 32 to specify the satisfaction with specialists, GPs and nurses. The NRS is a self-developed question with the following question “How satisfied are you with the received care on a 0 to 10 scale?”

Utilisation of paramedical care was assessed using the Medical Cost Questionnaire of the institute for Medical Technology Assessment (iMTA MCQ) [17]. The iMTA MCQ includes 31 questions and measures healthcare utilisation of the past 3 months (specific to the Dutch situation) [17]. The hypothesis is that there will be a shift of health care use to the primary care setting.

HrQoL was assessed using the EORTC-QoLC30, which is a validated questionnaire incorporating functional scales, a quality of life scale and symptom scales [18]. A QLQ-C30 summary score was calculated [19].

Mental health was assessed using the RAND Mental Health Inventory (MHI-5) including 5 items [20].

Self-efficacy was measured with three validated questionnaires [21–23]. The General Self-Efficacy Scale (GSE) assessed the self-belief to cope with a variety of difficult demands in life using 10 hypotheses [21]. The Perceived Efficacy in Patient-Physician Interactions (PEPPI-5) measures perceived self-efficacy [22]. The Pearlin Mastery Scale is designed to measure self-concept and the extent to which individuals perceive themselves in control of forces that significantly impact their lives [23]. For these three questionnaires, a higher scores indicates a higher self-efficacy.

### Adherence to the GRIP intervention

Adherence to the TOC was assessed using free text and coded information in the GPs’ EMR. The HON registered number and content of follow-up visits by using a personal plan and checklist. Data on collaboration between GP and HON was extracted from the GP’s EMR and based on data provided by the HON.

### Statistical analysis

Baseline characteristics were shown as means or medians for continuous variables and frequencies and percentages for categorical variables. Characteristics of patients who completed the study and patients who dropped-out were compared using independent T-tests for continuous variables and Pearson’s Chi-square used for categorical variables.

Intention-to-treat linear mixed regression analyses were used for continuous outcome variables adjusted for baseline variables (if measured at baseline) and treating hospital and cancer type. In these longitudinal analyses, the statistic model accounts for missing data based on the observed data [24]. For satisfaction with the specialist, GP and nurse T3 and T5 were analysed separately using an ANOVA adjusted for treating hospital and cancer type because only patients who had visited the corresponding healthcare workers received the questionnaire. The difference in healthcare utilisation for categorical data (i.e., paramedical care) was calculated with a Pearson Chi-Square or a Fisher exact test and count data with a log-binomial regression was dichotomized (i.e., no versus ≥1 ED visit), because the majority of patients had no visit. Healthcare utilisation outcomes were adjusted for treating hospital and cancer type. Additionally, because of group imbalances, we adjusted for co-morbidity (none/≥1) as sensitivity analysis.

Pre-specified subgroup analyses (co-morbidity (none/≥1), type of cancer (breast/colorectal/other), sex (male/female), age (≤65/> 65 year), baseline levels of the outcomes of interest) [11] were performed to explore differential intervention effects on patient satisfaction by adding interaction terms to the regression model.

## Results

**Fig. 2:**
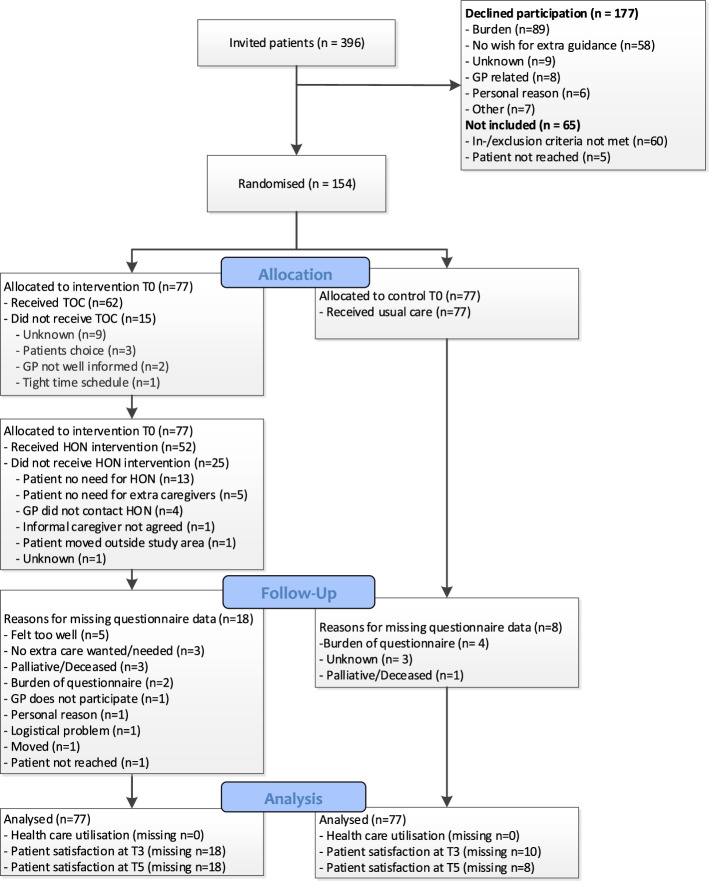
CONSORT flow diagram, Consolidated Standards of Reporting Trials of the GRIP study. Abbreviations: GP; General practitioner, HON; Home care Oncology Nurse, TOC; Time Out Consult

In total, 396 patients were invited and 154 (39%) patients actually participated in the GRIP trial, as shown in the CONSORT diagram (Fig. 2). These patients were registered with 119 different GPs, from 79 different primary care practices.

Information about the timing of T3 and T5 questionnaires within the cancer care pathway is provided in supplementary Additional file 1.

The mean age of the participants was 61 (SD ±11.9) years. The majority was female (75%) and had either breast (51%) or colorectal (25%) cancer (Table 1). The two study groups were comparable, except for a higher presence of comorbidity in the intervention group (68% versus 49%). Eighteen (23%) patients in the intervention group and 8 (10%) in the control group did not complete the T5 questionnaires. Characteristics of the analysed patients and the patients who dropped out did not differ (*p* > 0.05, Additional file 2).

**Table 1:** Characteristics of all study participants at baseline and missing study participants at T5

	Intervention *n =* 77	Intervention missing T5 *n =* 18 (23%)	Control *n =* 77	Control missing T5 *n =* 8 (10%)
Female n (%)	57 (74.0)	13 (72.2)	58 (75.3)	6 (75.0)
Age mean (±SD)	61.8 (11.4)	64 (9.5)	59.3 (12.2)	62 (11.9)
Cancer type n (%)				
Breast	38 (49.4)	8 (44.4)	40 (51.9)	4 (50.0)
Colorectal	20 (26.0)	6 (33.3)	18 (23.4)	1 (12.5)
Melanoma	13 (16.9)	2 (11.1)	11 (14.3)	1 (12.5)
Gynaecologic	3 (3.9)	2 (11.1)	2 (2.6)	2 (25.0)
Lung	3 (3.9)	–	6 (7.8)	–
Hospital setting n (%)				
Academic	22 (28.6)	7 (38.9)	24 (31.2)	2 (25)
Non academic	55 (71.4)	11 (61.1)	53 (68.8)	6 (75)
Cancer stage^1^				
0	2 (2.6)	–	2 (2.6)	–
I	34 (44.2)	6 (33.3)	34 (44.2)	5 (62.5)
II	22 (28.6)	5 (27.8)	27 (35.1)	3 (37.5)
III	18 (23.4)	6 (33.3)	14 (18.2)	–
IV	1 (1.3)	1 (5.6)	–	–
Education				
Low	32 (41.6)	9 (50)	25 (32.5)	1 (12.5)
Middle	13 (16.9)	1 (5.6)	18 (23.4)	4 (50.0)
High	32 (41.6)	8 (44.4)	34 (44.2)	3 (37.5)
Number of comorbidities n (%)				
None	25 (32.5)	4 (22.2)	39 (50.6)	2 (25.0)
> 1	52 (67.5)	14 (77.8)	38 (49.4)	6 (75.0)
Number of GP practice contacts (year prior inclusion) median (IQR)	7 (4.0;10.0)	7 (6.0;11.5)	6 (3.5;11.0)	9 (3.5;12.0)
GP years of working experience median (IQR)	17 (12.0;25.5)	17 (11.8;21.0)	16 (10.5;24.5)	18 (13.0;26.0)
GP setting n (%)				
Urban^2^	51 (66.2)	10 (55.6)	45 (58.4)	6 (75.0)
Between rural and urban^3^	14 (18.2)	3 (16.7)	15 (19.5)	2 (25.0)
Rural^4^	12 (15.6)	5 (27.8)	17 (22.1)	–

^1^Stage based on TNM classifications, ^2^1000 or more addresses per km^2, ^3^1000–1500 addresses per km^2, ^4^1000 or less addresses per km^2

Abbreviations: *SD* Standard deviation, *IQR* Inter quartile range

### Compliance with the GRIP intervention

Of the 77 intervention patients, 62 (81%) patients had a TOC [10]. Only 18% (*n* = 11) of these were scheduled according to the protocol, i.e., between diagnosis and treatment decision [10].

Fifty-two intervention patients (68%) had at least one contact with the HON. Reasons for not involving the HON were: no wish for HON involvement (*n* = 13, 17%) or no need for additional care providers (n^− 5^, 7%). Of the patients who had HON contact, 62% (*n* = 32) had three or more contact moments. The HON care was discontinued by 11 patients (18%) at their own request, after an average of three contacts. These patients either had an appointment to call for continuation of care but never called or they indicated to have enough support or feeling too well and no support was needed. HON contact was not continued after completion of active treatment by 24 patients (46%) who received HON guidance. Median number of contacts between the HON and GP was 2.0 (IQR 1;2). In the control group, no patient had a HON consultation.

### Patient satisfaction

#### Satisfaction with cancer care

Mean patient satisfaction with overall cancer care on the NRS (0–10 scale) did not differ between groups (T5: intervention 8.0 (SD ± 1.3), control 8.0 (SD ± 1.3)) (Table 2).

**Table 2:** Patient satisfaction with care scored on various themes - Overall care, specialist, general practitioner and nursing care assessment

		T3 measurement Mean (SD)		T5 measurement Mean (SD)	Between group^1^ Mean diff. (95% CI)	
OVERALL						
Information exchange					T3	T5
Intervention	*n =* 59	54.7 (21.5)	*n =* 59	54.2 (20.3)	−0.4 (−8.5;7.7)	1.4 (−6.7;9.4)
Control	*n =* 67	55.2 (22.8)	*n =* 69	53.3 (24.6)	Ref.	Ref.
Overall assessment					T3	T5
Intervention	*n =* 59	65.3 (21.3)	*n =* 59	66.1 (20.6)	1.9 (−5.8;9.7)	3.6 (−3.7;10.9)
Control	*n =* 67	63.8 (21.4)	*n =* 69	63.0 (21.3)	Ref.	Ref.
NRS (higher scores indicate better performance) 0–10 scale					T3	T5
Intervention	*n =* 59	8.1 (1.3)	*n =* 59	8.0 (1.3)	−0.1 (−0.5;0.4)	0.0 (−0.4;0.4)
Control	*n =* 67	8.2 (1.1)	*n =* 67	8.0 (1.3)	Ref.	Ref.
MEDICAL SPECIALIST						
Interpersonal skills – Specialist					T3	T5
Intervention	*n =* 59	69.9 (23.9)	*n =* 59	66.9 (23.9)	3.0 (−5.1;11.0)	4.8 (−2.8;12.5)
Control	*n =* 67	68.4 (22.6)	*n =* 69	62.8 (20.1)	Ref.	Ref.
Qualities - Specialist					T3	T5
Intervention	*n =* 59	79.2 (22.3)	*n =* 59	76.3 (22.9)	6.2 (−1.8;14.1)	3.9 (−3.9;11.6)
Control	*n =* 68	73.9 (23.0)	*n =* 69	73.6 (21.8)	Ref.	Ref.
Availability - Specialist					T3	T5
Intervention	*n =* 59	73.3 (21.2)	*n =* 59	69.9 (20.6)	3.3 (−4.4;11.1)	3.3 (−4.5;11.1)
Control	*n =* 67	70.1 (22.7)	*n =* 69	67.4 (23.6)	Ref.	Ref.
Relationship - Specialist					T3	T5
Intervention	*n =* 59	70.3 (22.5)	*n =* 59	71.2 (22.7)	5.7 (−2.6;14.0)	2.4 (−5.7;10.5)
Control	*n =* 68	65.4 (25.2)	*n =* 69	69.2 (22.7)	Ref.	Ref.
Tech. skills - Specialist					T3	T5
Intervention	*n =* 59	75.7 (19.0)	*n =* 59	72.6 (20.1)	4.2 (−2.4;10.8)	3.8 (− 2.9;10.5)
Control	*n =* 68	72.9 (20.3)	*n =* 69	69.6 (18.4)	Ref.	Ref.
Info. Provision - Specialist					T3	T5
Intervention	*n =* 59	70.3 (23.8)	*n =* 59	68.4 (21.6)	2.3 (−6.1;10.6)	3.7 (−3.6;11.0)
Control	*n =* 67	68.8 (22.7)	*n =* 69	65.1 (20.2)	Ref.	Ref.
GENERAL PRACTITIONER						
Interpersonal skills - GP					T3	T5
Intervention	*n =* 37	73.6 (24.3)	*n =* 38	63.2 (26.6)	2.6 (−11.5;16.6)	−9.6 (−22.6;3.3)
Control	*n =* 22	69.3 (26.9)	*n =* 31	70.7 (25.7)	Ref.	Ref.
Qualities - GP					T3	T5
Intervention	*n =* 37	78.4 (22.1)	*n =* 38	67.8 (25.9)	6.3 (−7.7;20.3)	−14.2 (−27.0;-1.3)
Control	*n =* 22	69.3 (29.8)	*n =* 31	79.8 (24.5)	Ref	Ref.
Availability - GP					T3	T5
Intervention	*n =* 37	75.7 (24.6)	*n =* 38	62.5 (26.5)	5.6 (−9.0;20.2)	−15.9 (−29.1;-2.6)
Control	*n =* 22	67.0 (29.3)	*n =* 31	75.0 (25.8)	Ref	Ref.
Relationship - GP					T3	T5
Intervention	*n =* 37	77.0 (22.3)	*n =* 38	70.4 (25.2)	8.2 (−5.7;22.1)	−6.2(−19.2;6.8)
Control	*n =* 22	67.0 (30.3)	*n =* 31	74.2 (27.0)	Ref	Ref.
Tech. skills - GP					T3	T5
Intervention	*n =* 37	66.9 (20.6)	*n =* 38	55.3 (21.9)	6.9 (−6,2;20.0)	−11.4 (−23.2;0.4)
Control	*n =* 22	59.5 (27.1)	*n =* 30	64.2 (24.1)	Ref	Ref.
Info. Provision - GP					T3	T5
Intervention	*n =* 37	60.4 (23.8)	*n =* 37	48.9 (24.9)	4.0 (−11.9;20.0)	−15.2 (−29.1;-1.4)
Control	*n =* 22	55.7 (31.2)	*n =* 29	60.6 (28.4)	Ref.	Ref.
NURSE ^2^						
Interpersonal skills - Nurse					T3	T5
Intervention	*n =* 33	72.7 (19.8)	*n =* 30	75.3 (20.9)	−0.3 (−11.5;10.8)	7.1 (−3.4;17.6)
Control	*n =* 30	73.6 (24.5)	*n =* 21	68.3 (21.3)	Ref.	Ref.
Experience/Knowl. - Nurse					T3	T5
Intervention	*n =* 33	68.2 (20.0)	*n =* 30	73.3 (20.7)	1.0 (−10.3;12.3)	10.8 (− 0.3;21.9)
Control	*n =* 30	67.5 (24.7)	*n =* 24	63.5 (22.1)	Ref.	Ref.
Availability -Nurse					T3	T5
Intervention	*n =* 33	74.2 (20.2)	*n =* 30	72.5 (23.1)	1.6 (−10.1;13.3)	7.7 (−3.7;19.0)
Control	*n =* 30	73.3 (26.2)	*n =* 22	65.9 (19.7)	Ref.	Ref.
Relationship-Nurse					T3	T5
Intervention	*n =* 33	68.2 (20.0)	*n =* 30	70.8 (22.8)	1.2 (−10.7;13.1)	1.2 (−9.6;12.1)
Control	*n =* 30	67.5 (26.4)	*n =* 24	70.8 (20.4)	Ref.	Ref.
Attention - Nurse					T3	T5
Intervention	*n =* 33	68.9 (20.8)	*n =* 30	75.8 (22.2)	0.2 (−11.9;12.4)	9.6 (−0.3;19.4)
Control	*n =* 30	69.2 (26.8)	*n =* 24	67.7 (20.2)	Ref.	Ref.
Willingness- Nurse					T3	T5
Intervention	*n =* 33	71.2 (21.8)	*n =* 30	75.8 (23.2)	1.4 (−10.7;13.5)	7.5 (−3.5;18.5)
Control	*n =* 30	70.8 (25.5)	*n =* 24	69.8 (20.8)	Ref.	Ref.

^1^Adjusted for stratification factors. ^2^Three themes not shown: Information disease, Information diagnostics and Information treatment

Abbreviations: *Diff* Difference, *GP* General practitioner, *Info* Information, *Knowl* Knowledge, *NRS* Number Rating Scale, *Ref* Reference group, *Tech* Technical

#### Satisfaction with care by GP

Between diagnosis and T3, 37 (48%) patients of the intervention group and 22 (29%) patients of the control group had received care from their GP. Among patients who visited their GP, satisfaction with GP care at T3 did not differ between the intervention and control group (Table 2).

From diagnosis till 3 months after treatment (T0-T5), 38 (49%) patients in the intervention group and 31 (40%) in the control group had received care from their GP. At T5, among these patients, patient satisfaction with GP care scores were significantly lower in the intervention group as compared to the control group on three subscales, i.e., Quality: between-group difference − 14.2 (95%CI -27.0;-1.3), Availability: -15.9 (95% CI -29.1;-2.6) and Information provision − 15.2 (95%CI -29.1;-1.4). Technical skills scored lower, bordering significance − 11.4 (95%CI -23.2;0.4) (Table 2).

#### Satisfaction with care by nurse

At T3, 33 (43%) patients of the intervention group and 30 (39%) patients of the control group had received care from a nurse. No difference was found in satisfaction with nursing care between groups. At T5, 30 (39%) patients of the intervention group and 24 (31%) of the control group had received care from a nurse. Patient satisfaction with nursing care concerning Experience/Knowledge, Availability, Attention and Willingness was - not significantly - higher in the intervention group compared to the control group (Table 2).

Explorative subgroup analyses suggest a non-significant, but potentially relevant higher satisfaction with overall care in patients with colorectal cancer in the intervention group compared to the control group (mean difference 9.9 (95% CI-3.8;23.6), Additional file 3). Intervention patients with colorectal cancer seems less satisfied with the GP as compared to the control group. Also, the subgroup analyses indicate that patients with ≥1 comorbidities, patients ≤65 years and female patients scored lower on several GP satisfaction subscales (Additional file 3). In contrast, nurse satisfaction score was lower among patients with colorectal cancer and breast cancer compared to the other cancer types, in patients without comorbidities, in patients > 65 years and female patients.

#### Healthcare utilisation

The intervention group had a significantly higher “risk” of having contact with the GP practice (RR: 1.3 (95% CI 1.0;1.7) *p* = 0.03) and ED visits (RR: 1.7 (95% CI 1.0;2.8) *p* = 0.04) compared to the control group (Table 3). After adjustment for co-morbidity, RRs were 1.3 (95% CI 0.994;1.603) for contact with GP practice and 1.9 (95% CI 1.01;3.45) for ED visits. No other significant between- group differences in use of hospital or paramedical care were found (Table 3).

**Table 3:** Health care utilization in primary care and hospital 1 year after inclusion and paramedical care 3 months before T5 assessment

Health care utilization primary care 1 year after inclusion			
	Intervention *n =* 77 Median (IQR)	Control *n =* 77 Median (IQR)	Negative binomial regression RR (95% CI)
Contacts with GP practice (incl. Out of office hours)	9 (5.0;16.0)	8 (5.0;13.5)	1.3 (1.0;1.7)*
Contacts with GP (incl. Out of office hours)	7 (5.0;12.0)	6 (4.0;9.5)	1.3 (1.0;1.6)
Contacts with GP (excl. Out of office hours)	7 (4.5;12.0)	6 (4.0;9.5)	1.2 (1.0;1.5)
**Health care utilization hospital care 1 year after inclusion**			
Total contacts (incl. by phone + consultations + ED + hospitalizations+ diagnostics)	49 (27.5;88.5)	50 (24.5;78.5)	1.2 (1.0;1 .4)
Contacts by phone	6 (3.0;14.5)	7 (3.0;13.0)	1.2 (0.9;1.5)
Consultations	20 (11.5;31.0)	20 (13.0;30.0)	1.0 (0.9;1.2)
	n (%)	n (%)	RR^1^ (95% CI).
Patients visiting the ED	29 (37.7)	17 (22.1)	1.7 (1.0;2.8)**
Patients with emergency hospitalizations	13 (16.9)	9 (11.7)	1.5 (0.7;3.2)
Health care utilisation paramedical care in the 3 months before T5.			
	Intervention *n =* 59	Control *n =* 69	*p-*value
	n (%)	n (%)	
Physiotherapy total	29 (49.2)	35 (50.7)	0.86
- in primary care	22 (75.9)	29 (82.9)	
- in hospital	1 (3.4)	2 (5.7)	
- both	6 (20.7)	4 (11.4)	
Ergo therapy	1 (1.7)	2 (2.9)	1.00
- in hospital	1 (100)	2 (100)	
Acupuncture/homeopathy	3 (5.1)	3 (4.3)	1.00
- in primary care	3 (100)	3 (100)	
Psychologist	10 (16.9)	10 (14.5)	0.70
- in primary care	6 (60.0)	7 (70.0)	
- in hospital	2 (20.0)	1 (10.0)	
- both	2 (20.0)	2 (20.0)	
Dietician	2 (3.4)	3 (4.3)	1.00
- in primary care	1 (50.0)	2 (66.7)	
- in hospital	1 (50.0)	1 (33.3)	
Speech therapist	–	–	–

*p-value = 0.03 **p-value = 0.04. ^1^Adjusted for stratification factors

Abbreviations: *ED* Emergency Department, *GP* General practitioner, *RR* Relative risk, *Int* Intervention, *Cont* Control

#### Secondary outcomes

No significant between-group differences were found for global QoL, the QoL-Summary scale, mental health and self-efficacy (Table 4 and Table 5).

**Table 4:** Quality of life at T5

	Intervention (*N =* 59) Mean (SD)	Control (*N =* 69) Mean (SD)	Between group^1^ - Mean diff. (95% CI)
Function scales			
Physical Function	78.8 (22.1)	81.5 (18.4)	−0.5 (−6.0;4.9)
Role Function	70.6 (31.0)	75.4 (28.4)	−4.6 (− 14.7;5.5)
Emotional Function	80.4 (22.6)	79.8 (23.5)	−1.7 (−9.4;6.1)
Cognitive Function	79.4 (23.8)	77.3 (24.9)	3.2 (− 4.7;11.2)
Social Function	79.7 (25.0)	78.7 (28.9)	−1.2 (− 10.6;8.1)
Symptom scales			
Fatigue	31.3 (27.2)	32.9 (27.4)	−2.0 (−10.8;6.9)
Nausea/vomiting	2.8 (9.9)	2.9 (8.1)	−0.2 (−3.4;3.0)
Pain	23.7 (30.5)	20.5 (24.4)	2.1 (−7.0;11.3)
Single items			
Dyspnoea	13.6 (24.9)	12.1 (24.9)	2.2 (−4.1;8.5)
Insomnia	30.5 (34.1)	28.0 (31.1)	2.2 (−8.0;12.4)
Appetite loss	4.0 (12.5)	8.2 (20.9)	−4.4 (−10.1;1.3)
Constipation	10.2 (25.0)	5.8 (16.1)	2.7 (−4.5;9.8)
Diarrhoea	5.7 (19.7)	7.7 (19.9)	−1.6 (−8.7;5.5)
Financial difficulties	7.9 (17.9)	11.6 (24.1)	−4.1 (−11.7;3.6)
Global scales			
Global Quality of life	71.9 (19.1)	72.6 (20.5)	−1.2 (−7.6;5.3)
Summary functioning scale	82.1 (17.0)	82.7 (15.5)	−0.4 (−5.4;4.6)

^1^ Adjusted for stratification factors and baseline

Abbreviations: *CI* Confidence Interval, *SD* Standard Deviation

**Table 5:** T5 Secondary outcomes – Mental health and Self-efficacy

	Intervention *n =* 59 mean (SD)	Control *n =* 69 mean (SD)	Mean difference (95% CI)	Mean difference^1^ (95% CI)
MHI-5 (> 60 score indicate mentally healthy) 0–100 scale.	75.1 (15.7)	73.6 (17.2)	1.6 (−4.2; 7.4)	− 0.6 (−6.0; 4.9)
Pearlin-Schooler Mastery scale (higher scores indicate better performance) 5–35 scale.	25.0 (4.9)	24.8 (4.2)	0.2 (−1.4; 1.8)	− 0.0 (− 1.6;1.6)
GSE (higher scores indicate better performance) 10–40 scale.	32.3 (4.1)	31.3 (4.2)	1.0 (− 0.5; 2.4)	0.3 (− 1.0;1.5)
PEPPI (higher scores indicate better performance) 5–25 scale.	20.7 (3.3)	21.4 (3.0)	−0.8 (−1.8; 0.2)	− 0.6 (− 1.4;0.3)

^1^ Adjusted for stratification factors and baseline value of the outcome

Abbreviations: *CI* Confidence Interval, *SD* Standard Deviation

## Discussion

All cancer patients in our study reported high satisfaction with care, independent whether they received specialist care alone or additional structured care from a GP and HON. Although the GRIP intervention was designed to improve primary care involvement, the ability to measure its effectiveness was hampered because it was often not implemented as intended: 82% of the TOCs were not planned before the treatment decision and 46% of patients receiving HON care did not continue after treatment completion. In our trial, structured involvement of primary care during cancer treatment did not result in increased patient satisfaction, nor did it improve HrQoL, mental health or self-efficacy. Additional guidance from primary care did result in more ED visits.

Patients seem well motivated to actively involve their primary care team, since the intervention uptake was relatively high; 81% of patients in the intervention group scheduled a TOC and 68% had HON consultations. Other studies investigating primary care involvement reported a lower uptake, varying from 27 to 60% [25–27]. The high uptake of primary care involvement is in line with earlier reports of the Dutch Cancer patients organisation [8], which demonstrated a strong wish for more GP involvement among cancer patients.

In contrast with our initial hypothesis, patients in the intervention group were less satisfied with their GP and slightly more with their nurse. This may be explained in several ways. First, the intervention itself may have raised expectations about GP involvement in the intervention group; which were not met in practice. Patients receiving the intervention were notified that they would receive extra care from the primary care team: both their GP and a HON. They might have expected more contact with their GP but met the HON instead. The significantly lower scores on “GP-Availability” in the intervention group support this hypothesis. Another possible explanation may be that in the control group, all GP involvement was the result of an independent proactive approach by the GP, which might occur more often in case of a better patient-GP relationship. In the intervention group, GP involvement was started by protocol, potentially leading to GP contacts with patients with a lower ‘baseline satisfaction’ with their GP.

Finally, the lack of difference in satisfaction with care may be the result of a ceiling effect, which is supported by the high overall satisfaction scores in both study groups. In the Netherlands, patients usually have a nurse as case manager in the hospital, which might contribute to the high satisfaction.

Other studies evaluating primary care interventions after cancer diagnosis in the curative patient population indicate either positive effects on patient satisfaction [25, 27, 28] or no effects [29] and showed less ED visits in the older population [30]. These studies examined various interventions, which involved information provision using patient health records [25, 27, 28] or intensified primary care with the focus on GP [27] or on a primary care team [30]. The variety of interventions, different healthcare systems, or the use of self-developed questionnaires to measure patient satisfaction [25, 27, 28, 31] might explain the more positive outcome as compared to our study.

The higher number of ED visits among intervention patients compared to the control group was in contrast with our expectations. Reasons for the observed ED consultations were mostly oncology-related and seemed unavoidable, e.g., ED visits because of fever during chemotherapy. Although the ED records did not provide clues for the reasons for increased ED use in the intervention group, it may be related to the fact that GPs referred cancer patients at a lower threshold because of study participation. Another explanation might be that patients in the intervention group had more comorbidity. However, adjustment only slightly affected the estimates.

### Study limitations

This study has both strengths and limitations. The main strength is the pragmatic approach and the implementation of the study in daily practice. Consequently, this pragmatic RCT adds to the scarce evidence on the real-life effects of involving a primary care team during and after curative cancer treatment. Also, outcome measurement schedules were aligned to individual patient’s cancer treatments, thereby enabling different cancer types to be included.

Present study results might not be generalizable to all cancer patients who are to be treated with curative intent, since our study population might be a selection of patients who were positive towards primary care. Also, our intervention may have been prematurely implemented, as supported by the relatively high uptake but incorrect scheduling (TOC) and relatively high number of discontinued HON contacts. We recommend future studies to follow strategies to develop and evaluate a complex intervention as presented in the framework of the Medical Research Council [32] more strictly. This approach would require more elaborate pilot evaluations to optimize the individual elements of the intervention, the intervention procedures and to optimize the definition and assessments of outcomes.

Another limitation was that patients and healthcare providers could not be blinded, due to the nature of the intervention, which may have affected outcomes. Furthermore, several patients stopped study participation and we found a higher drop-out in the intervention group compared to the control group. Even though patients’ characteristics of drop-outs did not differ, this might have caused selection bias.

### Implications for practice

The present study showed that the majority of patients was motivated to involve the GP after their cancer diagnosis. Unfortunately, high uptake was followed by suboptimal implementation of the intended intervention and, in its current form’ it did not result in improved satisfaction. Therefore, adjustment of the design and/or implementation of the intervention is required. The design and content of the HON intervention may not have matched the needs of almost half of the patients. Possibly, the patients expected their GP to be personally involved, and not to delegate care to the HON. This deserves further exploration.

## Conclusions

The GRIP intervention, which aimed to structure involvement of primary care during and after cancer treatment with curative intent, was well accepted but sub-optimally implemented and adhered to. It slightly increased primary healthcare contacts, did not improve patient satisfaction with care and increased use of the ED. As the high uptake of the intervention suggests that it addresses patients’ needs, future research should first focus on optimizing the design and implementation of primary care involvement. This future effort may benefit from an integrated and collaborative approach with patients and healthcare professionals.

## Supplementary Information

**Additional file 1.**

**Additional file 2.**

**Additional file 3.**

## Data Availability

The original datasets generated and/or analysed during the current study are not publicly available due to privacy restrictions, anonymised analysis datasets are available from the corresponding author on reasonable request.

## Abbreviations

ED: Emergency Department
EMR: Electronic Medical Record
EORTC-INPATSAT-32: European Organisation for Research and Treatment of Cancer Satisfaction with care questionnaire
EORTC-QoLC30: European Organisation for Research and Treatment of Cancer Quality of Life questionnaire
GP: General practitioner
GSE: General Self-Efficacy Scale
HON: Home care Oncology Nurse
hrQoL: Health related quality of life
iMTA MCQ: Medical Cost Questionnaire of the institute for Medical Technology Assessment
IQR: Inter quartile range
MHI-5: RAND Mental Health Inventory
NRS: Numeric Rating Scale
PEPPI-5: Perceived Efficacy in Patient-Physician Interactions
RCT: Randomised controlled trial
SDM: Shared decision making
T0: Timing of baseline assessment
T1: Timing of questionnaire assessment 2 weeks after inclusion
T2: Timing of questionnaire assessment 3 months after inclusion
T3: Timing of questionnaire assessment 6 months after inclusion or if primary treatment was completed within 6 months after inclusion, T3 was planned at completion of treatment.
T4: Timing of questionnaire assessment 9 months after inclusion
T5: Timing of questionnaire assessment 12 months after inclusion or if primary treatment was completed within 6 months after inclusion, T3 was planned at completion of treatment and T5 3 months later
TOC: Time Out Consult
