# Supplementary material for: Effects of structured involvement of the primary care team versus standard care after a cancer diagnosis on patient satisfaction and healthcare use: the GRIP randomised controlled trial
Source: BMC Prim Care. 2022 Jun 4;23:145. doi: 10.1186/s12875-022-01746-3 (PMC9166421; doi:10.1186/s12875-022-01746-3)
Supplement: Supplementary file 1 — Additional file 1. [file 12875_2022_1746_MOESM1_ESM.docx]

**Additional file 1.** Timing of questionnaires during- and after cancer treatment

|  |
| --- |

**Figure A.** Study time frame of patients filled out T5 – per cancer type. Mean number of days between inclusion and assessed questionnaires: T3 and T5.

Patients received the T3 questionnaire when they completed active treatment or 6 months after inclusion. Patients received the T5 questionnaire 3 months after primary treatment or 12 months after inclusion. In both study groups the majority of patients (69%) completed primary treatment at T3 (n=41/59 in the intervention group and n= 46/67 in the control group). At T5 one patient still received primary treatment (control). The median number of days between completion of active treatment and T5 was 94 (IQ 90;100) for the intervention group and 93 (IQR 90;102.5) for the control group. The timing of questionnaires in the varying cancer treatment schedules is shown in figure 2. In the intervention group, the median time between T0 and T3 was 134 days (IQR 72-183) and from T0 to T5: 209 days (IQR 150-332). For the control group, the T3 median duration was 155 days (IQR 71-186) and T0 to T5 was 219 days (IQR 147-309).
