# Supplementary material for: Effects of structured involvement of the primary care team versus standard care after a cancer diagnosis on patient satisfaction and healthcare use: the GRIP randomised controlled trial
Source: BMC Prim Care. 2022 Jun 4;23:145. doi: 10.1186/s12875-022-01746-3 (PMC9166421; doi:10.1186/s12875-022-01746-3)
Supplement: Supplementary file 2 — Additional file 2. [file 12875_2022_1746_MOESM2_ESM.docx]

**Additional file 2.** Baseline extended.

**Table B.** Overview of patient characteristics at baseline and at T5 the analysed and missing population.

|  | **Intervention**  **N = 77** | Intervention  Analysis T5  N=59 (77%) | Intervention  Missing T5  N=18 (23%) | **Control**  **N = 77** | Control  Analysis T5  N= 69 (90%) | Control  Missing T5  N=8 (10%) |
| --- | --- | --- | --- | --- | --- | --- |
| **Female N (%)** | 57 (74.0) | 44 (74.6) | 13 (72.2) | 58 (75.3) | 52 (75.4) | 6 (75.0) |
| **Age mean (±SD)** | 61.8 (11.4) | 61 (11.9) | 64 (9.5) | 59.3 (12.2) | 59 (12.3) | 62 (11.9) |
| **Cancer type N (%)**  Breast  Colorectal  Melanoma  Gynaecologic  Lung | 38 (49.4)  20 (26.0)  13 (16.9)  3 (3.9)  3 (3.9) | 30 (50.8)  14 (23.7)  11 (18.6)  1 (1.7)  3 (5.1) | 8 (44.4)  6 (33.3)  2 (11.1)  2 (11.1)  - | 40 (51.9)  18 (23.4)  11 (14.3)  2 (2.6)  6 (7.8) | 36 (52.2)  17 (24.6)  10 (14.5)  4 (5.8)  2 (2.9) | 4 (50.0)  1 (12.5)  1 (12.5)  2 (25.0)  - |
| **Hospital setting N (%)**  Academic  Non academic | 22 (28.6)  55 (71.4) | 15 (25.4)  44 (74.6) | 7 (38.9)  11 (61.1) | 24 (31.2)  53 (68.8) | 22 (31.9)  47 (68.1) | 2 (25)  6 (75) |
| **Cancer stage^1^**  0  I  II  III  IV | 2 (2.6)  34 (44.2)  22 (28.6)  18 (23.4)  1 (1.3) | 2 (3.4)  28 (47.5)  17 (28.8)  12 (20.3)  - | -  6 (33.3)  5 (27.8)  6 (33.3)  1 (5.6) | 2 (2.6)  34 (44.2)  27 (35.1)  14 (18.2)  - | 2(2.9)  29 (42.0)  24 (34.8)  14 (20.3)  - | -  5 (62.5)  3 (37.5)  -  - |
| **Education**  Low  Middle  High | 32 (41.6)  13 (16.9)  32 (41.6) | 23 (39.0)  12 (20.3)  24 (40.7) | 9 (50)  1 (5.6)  8 (44.4) | 25 (32.5)  18 (23.4)  34 (44.2) | 24 (34.8)  14 (20.3)  31 (44.9) | 1 (12.5)  4 (50.0)  3 (37.5) |
| **Number of comorbidities N (%)**  None  >1 | 25 (32.5)  52 (67.5) | 21 (35.6)  38 (64.4) | 4 (22.2)  14 (77.8) | 39 (50.6)  38 (49.4) | 37 (53.6)  32 (46.4) | 2 (25.0)  6 (75.0) |
| **Number of GP practice contacts (yr. prior inclusion) median (IQR)** | 7 (4.0;10.0) | 6 (3.0;10.0) | 7 (6.0;11.5) | 6 (3.5;11.0) | 6 (3.5;11.0) | 9 (3.5;12.0) |
| **GP yrs of working experience median (IQR)** | 17 (12.0;25.5) | 17 (12.0;28.0) | 17 (11.8;21.0) | 16 (10.5;24.5) | 16 (10.0;24.5) | 18 (13.0;26.0) |
| **GP setting N (%)**  Urban^2^  Between rural and urban^3^  Rural^4^ | 51 (66.2)  14 (18.2)  12 (15.6) | 41 (69.5)  11 (18.6)  7 (11.9) | 10 (55.6)  3 (16.7)  5 (27.8) | 45 (58.4)  15 (19.5)  17 (22.1) | 39 (56.5)  13 (18.8)  17 (24.6) | 6 (75.0)  2 (25.0)  - |

Baseline characteristics between the drop-out and analysed group at T5 did not differ.

^1^ Stage based on TNM classifications, ^2^ 31000 or more addresses per km^2, ^3^ 1000-1500 addresses per km^2, ^4^ 1000 or less addresses per km^2)

Abbreviations: SD; Standard deviation, IQR; Inter quartile range, yr; year.
