# Supplementary material for: Effects of structured involvement of the primary care team versus standard care after a cancer diagnosis on patient satisfaction and healthcare use: the GRIP randomised controlled trial
Source: BMC Prim Care. 2022 Jun 4;23:145. doi: 10.1186/s12875-022-01746-3 (PMC9166421; doi:10.1186/s12875-022-01746-3)
Supplement: Supplementary file 3 — Additional file 3. [file 12875_2022_1746_MOESM3_ESM.docx]

**Additional file 3.** Subgroup analysis – Patient Satisfaction with care.

**Table C1.** Subgroup effects on overall patient satisfaction with care.

| Overall Satisfaction | N int./  N cont. | **T3 estimated mean difference int. vs cont. (95% CI)** | **p value** | N int./  N cont. | **T5 estimated mean difference int. vs cont. (95% CI)** |
| --- | --- | --- | --- | --- | --- |
| **Total effects** | 59/67 | 1.9 (-5.8;9.7) |  | 59/69 | 3.6 (-3.7;10.9) |
| **Sexe**  Male  Female | 15/17  44/50 | -0.2 (-15.7;15.2)  2.2 (-7.2;11.5) | 0.78 | 15/17  44/52 | -2.9 (-15.9;10.2)  4.8 (-4.2;13.9) |
| **Type of cancer**  Breast  Colorectal  Other | 28/34  16/16  15/17 | 1.7 (-8.8;12.2)  10.9 (-5.5;27.4)  -8.2 (-25.5;9.1) | 0.28 | 30/36  14/17  15/16 | 4.3 (-6.6;15.3)  9.9 (-3.8;23.6)  -7.7 (-23.1;7.6) |
| **Age**  ≤65  >65 | 37/46  22/21 | 5.4 (-5.1;15.9)  -0.8 (-10.9;9.3) | 0.52 | 36/47  23/22 | 5.7 (-3.8;15.2)  4.3 (-7.4;16.0) |
| **Comorbidity**  None  ≥1 | 21/35  38/32 | -1.0 (-13.4;11.4)  5.9 (-4.5;16.2) | 0.50 | 21/37  38/32 | 0.7 (-11.8;13.1)  5.7 (-3.7;15.2) |

Abbreviation: int; intervention group, cont; control group.

**Table C2.** Subgroup effects on patient satisfaction with general practitioner’s interpersonal skills scale.

| Interpersonal skills - GP | N int./  N cont. | **T3 estimated mean difference int. vs cont. (95% CI)** | **p value** | N int./  N cont. | **T5 estimated mean difference int. vs cont. (95% CI)** |
| --- | --- | --- | --- | --- | --- |
| **Total effects** | 37/22 | 2.0 (-12.2;16.2) |  | 38/31 | -8.6 (-21.2;4.0) |
| **Sexe**  Male  Female | 8/8  29/14 | 11.3 (-10.0;32.7)  1.7 (-19.8;23.3) | 0.67 | 8/12  30/19 | -0.4 (-22.5; 23.2)  -7.5 (-25.7;10.7) |
| **Type of cancer**  Breast  Colorectal  Other | 17/13  11/5  9/4 | -1.7 (-23.7; 20.3)  0.4 (-20.4;21.1)  13.4 (-21.7;48.5) | 0.95 | 20/14  9/11  9/6 | -4.8 (-27.0;17.4)  -22.3 (-43.2;2.5)  -8.7 (-29.9;12.6) |
| **Age**  ≤65  >65 | 25/12  12/10 | 3.5 (-15.6;22.5)  7.6 (-11.8;26.9) | 0.35 | 24/19  14/12 | -6.8 (-22.6;9.0)  -7.8 (-29.0;13.5) |
| **Comorbidity**  None  ≥1 | 16/11  21/11 | 16.0 (-17.5;49.5)  -2.6 (-19.1;13.9) | 0.43 | 13/15  25/16 | 3.2 (-23.1;29.6)  -13.6 (-29.6;2.4) |

Abbreviation: GP; general practitioner, int; intervention group, cont; control group.

**Table C3.** Subgroup effects on patient satisfaction with general practitioner’s quality.

| Qualities - GP | N int./  N cont. | **T3 estimated mean difference int. vs cont. (95% CI)** | **p value** | N int./  N cont. | **T5 estimated mean difference int. vs cont. (95% CI)** |
| --- | --- | --- | --- | --- | --- |
| **Total effects** | 37/22 | 6.0 (-7.8;19.8) |  | 38/31 | -13.8 (-26.2;-1.3) |
| **Sexe**  Male  Female | 8/8  29/14 | 10.8 (-11.8;33.5)  7.3 (-13.0;27.7) | 0.87 | 8/12  30/19 | -7.6 (-35.0;19.8)  -11.3 (-28.4;5.7) |
| **Type of cancer**  Breast  Colorectal  Other | 17/13  11/5  9/4 | 4.7 (-17.8;27.3)  0.1 (-22.0;22.3)  18.8 (-6.6;44.1) | 0.85 | 20/14  9/11  9/6 | -7.2 (-26.9;12.5)  -23.7 (-49.0;1.5)  -20.5 (-47.8;6.9) |
| **Age**  ≤65  >65 | 25/12  12/10 | 4.5 (-15.0;23.9)  15.4 (0.7;30.1) | 0.13 | 24/19  14/12 | -16.5 (-32.8;-0.3)  -5.1 (-26.1;15.8) |
| **Comorbidity**  None  ≥1 | 16/11  21/11 | 18.2 (-14.9;51.3)  2.6 (-13.3;18.5) | 0.49 | 13/15  25/16 | -8.2 (-33.1;16.6)  -15.8 (-32.9;1.2) |

Abbreviation: GP; general practitioner, int; intervention group, cont; control group.

**Table C4.** Subgroup effects on patient satisfaction with general practitioner’s availability.

| Availability - GP | N int./  N cont. | **T3 estimated mean difference int. vs cont. (95% CI)** | **p value** | N int./  N cont. | **T5 estimated mean difference int. vs cont. (95% CI)** |
| --- | --- | --- | --- | --- | --- |
| **Total effects** | 37/22 | 5.0 (-9.7;19.8) |  | 38/31 | -14.3 (-27.4;-1.3) |
| **Sexe**  Male  Female | 8/8  29/14 | 13.5 (-9.8;36.8)  4.6 (-17.8;26.9) | 0.67 | 8/12  30/19 | 5.4 (-18.0;28.9)  -16.5 (-34.4;1.4) |
| **Type of cancer**  Breast  Colorectal  Other | 17/13  11/5  9/4 | 2.3 (-22.0;26.5)  0.1 (-22.0;22.3)  21.7 (-8.7;52.2) | 0.79 | 20/14  9/11  9/6 | -14.1 (-35.9;7.70  -16.9 (-42.7;8.8)  -15.6 (-39.7;8.5) |
| **Age**  ≤65  >65 | 25/12  12/10 | 4.0 (-16.9;24.8)  13.3 (-7.4;33.9) | 0.24 | 24/19  14/12 | -12.2 (-29.4;4.9)  -16.2 (-37.3;5.0) |
| **Comorbidity**  None  ≥1 | 16/11  21/11 | 17.2 (-16.2;50.7)  1.4 (-17.0;19.9) | 0.53 | 13/15  26/16 | -4.9 (-28.6;18.8)  -16.6 (-34.6;1.5) |

Abbreviation: GP; general practitioner, int; intervention group, cont; control group.

**Table C5.** Subgroup effects on patient satisfaction with their general practitioner relationship.

| Relationship – GP | N int./  N cont. | **T3 estimated mean difference int. vs cont. (95% CI)** | **p value** | N int./  N cont. | **T5 estimated mean difference int. vs cont. (95% CI)** |
| --- | --- | --- | --- | --- | --- |
| **Total effects** | 37/22 | 7.1 (-6.9;21.1) |  | 38/31 | -5.3 (-18.2;7.5) |
| **Sexe**  Male  Female | 8/8  29/14 | 15.5 (-11.3;42.2)  10.9 (-9.6;31.3) | 0.92 | 8/12  30/19 | -5.4 (34.1;23.2)  -1.5 (-19.2;16.1) |
| **Type of cancer**  Breast  Colorectal  Other | 17/13  11/5  9/4 | 9.2 (-12.8;31.1)  3.1 (-21.3; 27.4)  14.9 (-14.7;44.6) | 0.99 | 20/14  9/11  9/6 | 1.5 (-20.1;23.2)  -14.8 (-41.1;11.4)  -16.9 (-33.9;0.1) |
| **Age**  ≤65  >65 | 25/12  12/10 | 11.3 (-8.0;30.5)  13.1 (-5.6;31.8) | 0.37 | 24/19  14/12 | -6.9 (-23.7;10.0)  3.7 (-14.6;22.0) |
| **Comorbidity**  None  ≥1 | 16/11  21/11 | 15.3 (-16.5;47.1)  6.2 (-11.6;24.1) | 0.90 | 13/15  25/16 | -1.7 (-27.7;24.2)  -4.5 (-22.4;13.3) |

Abbreviation: GP; general practitioner int; intervention group, cont; control group.

**Table C6.** Subgroup effects on patient satisfaction with the general practitioner’s technical skills.

| Tech. skills - GP | N int./  N cont. | **T3 estimated mean difference int. vs cont. (95% CI)** | **p value** | N int./  N cont. | **T5 estimated mean difference int. vs cont. (95% CI)** |
| --- | --- | --- | --- | --- | --- |
| **Total effects** | 37/22 | 5.9 (-7.4;19.2) |  | 38/30 | -9.9 (-21.6;1.7) |
| **Sexe**  Male  Female | 8/8  29/14 | 11.3 (-10.0;32.5)  7.4 (-12.9;27.8) | 0.96 | 8/11  30/19 | 1.6 (-19.7;22.9)  -11.4 (-27.9;5.1) |
| **Type of cancer**  Breast  Colorectal  Other | 17/13  11/5  9/4 | 5.8 (-16.6;28.2)  0.2 (-20.2;20.5)  16.5 (-7.1;40.0) | 0.90 | 20/14  9/10  9/6 | -10.3 (-30.5;10.0)  -17.2 (36.9;2.4)  -9.1 (-32.1;13.9) |
| **Age**  ≤65  >65 | 25/12  12/10 | 9.4 (-10.0;28.9)  7.9 (-10.0;25.9) | 0.68 | 24/18  14/12 | -8.3 (-23.8;7.3)  -9.6 (-27.2;7.9) |
| **Comorbidity**  None  ≥1 | 16/11  21/11 | 13.9 (-14.7;42.5)  2.5 (-15.0;20.1) | 0.48 | 13/15  25/15 | -5.5 (-27.7;16.6)  -12.0 (-27.7;3.8) |

Abbreviation: GP; general practitioner, int; intervention group, cont; control group.

**Table C7.** Subgroup effects on patient satisfaction with information provision from their general practitioner.

| Info. provision - GP | N int./  N cont. | **T3 estimated mean difference int. vs cont. (95% CI)** | **p value** | N int./  N cont. | **T5 estimated mean difference int. vs cont. (95% CI)** |
| --- | --- | --- | --- | --- | --- |
| **Total effects** | 37/22 | 3.8 (-11.8;19.4) |  | 37/29 | -13.2 (-26.9;0.5) |
| **Sexe**  Male  Female | 8/8  29/14 | 6.9 (-21.5;35.3)  6.8 (-16.4;30.0) | 0.87 | 8/11  29/18 | -1.4 (-19.2;22.0)  -13.1 (-33.4;7.2) |
| **Type of cancer**  Breast  Colorectal  Other | 17/13  11/5  9/4 | 3.2 (-23.4;29.7)  -3.9 (-30.9;23.2)  10.7 (-14.5;23.9) | 0.89 | 19/13  9/10  9/6 | -16.0 (-41.4;9.4)  -18.8 (-40.8;3.3)  -9.7 (-33.5;14.0) |
| **Age**  ≤65  >65 | 25/12  12/10 | 8.8 (-13.9;31.4)  4.9 (-17.5;27.3) | 0.76 | 24/18  13/11 | -11.7 (-30.5;7.1)  -8.3 (-25.7;9.0) |
| **Comorbidity**  None  ≥1 | 16/11  21/11 | 5.6 (-28.9;40.0)  -1.4 (-20.8;18.1) | 0.59 | 13/15  24/14 | -9.1 (-36.1;17.9)  -14.1 (-33.6;5.5) |

Abbreviation: GP; general practitioner, int; intervention group, cont; control group.

**Table C8.** Subgroup effects on patient satisfaction with nurse’s interpersonal skills.

| Interpersonal skills - Nurse | N int./  N cont. | **T3 estimated mean difference int. vs cont. (95% CI)** | **p value** | N int./  N cont. | **T5 estimated mean difference int. vs cont. (95% CI)** |
| --- | --- | --- | --- | --- | --- |
| **Total effects** | 33/30 | -0.3 (-11.5;10.8) | 0.95 | 30/21 | 7.1 (-3.4;17.6) |
| **Sexe**  Male  Female | 7/7  26/23 | -2.0 (-29.6;25.6)  2.9 (-10.3;16.0) | 0.88 | 7/6  23/15 | 11.2 (-14.3;36.7)  6.5 (-5.6;18.6) |
| **Type of cancer**  Breast  Colorectal  Other | 17/18  11/6  5/6 | -3.4 (-17.4;10.6)  9.3 (-18.3;37.0)  -10.5 (-31.8;10.7) | 0.98 | 18/12  8/7  4/2 | -0.1 (-13.7;13.6)  13.4 (-8.0;34.9)  28.6 (16.0;41.1) |
| **Age**  ≤65  >65 | 21/20  12/10 | 3.2 (-11.9;18.2)  -6.8 (-22.8;9.2) | 0.88 | 20/13  10/8 | 11.6 (-1.0;24.1)  -5.5 (-20.5;9.4) |
| **Comorbidity**  None  ≥1 | 12/18  21/12 | -4.3 (-22.3;13.6)  1.3 (-16.3;18.8) | 0.77 | 11/11  19/10 | 1.4 (-18.1;20.8)  12.2 (-3.5;27.8) |

Abbreviation: int; intervention group, cont; control group.

**Table C9.** Subgroup effects on patient satisfaction with nurse’s knowledge and experience.

| Knowledge & Experience - Nurse | N int./  N cont. | **T3 estimated mean difference int. vs cont. (95% CI)** | **p value** | N int./  N cont. | **T5 estimated mean difference int. vs cont. (95% CI)** |
| --- | --- | --- | --- | --- | --- |
| **Total effects** | 33/30 | 1.0 (-10.3;12.3) | 0.86 | 30/24 | 10.8 (-0.3;21.9) |
| **Sexe**  Male  Female | 7/7  26/23 | 10.3 (-16.4;37.0)  1.2 (-12.1;14.6) | 0.42 | 7/6  23/18 | 14.8 (-10.4;40.0)  10.4 (-2.4;23.2) |
| **Type of cancer**  Breast  Colorectal  Other | 17/18  11/6  5/6 | -3.6 (-18.1;11.0)  13.7 (-12.3;39.6)  -11.7 (-38.5;15.0) | 0.81 | 18/14  8/7  4/3 | 6.0 (-8.7;20.7)  7.5 (-16.4;31.3)  41.7 (20.0;63.3) |
| **Age**  ≤65  >65 | 21/20  12/10 | 3.1 (-13.4;19.6)  -1.7 (-16.5;13.1) | 0.91 | 20/16  10/8 | 13.3 (-0.7;27.3)  2.1 (-15.5;19.7) |
| **Comorbidity**  None  ≥1 | 12/18  21/12 | 1.2 (-19.7;22.0)  -2.9 (-18.9;13.0) | 0.73 | 11/14  19/10 | 1.1 (-17.3;19.6)  13.8 (-2.8;30.4) |

Abbreviation: int; intervention group, cont; control group.

**Table C10.** Subgroup effects on patient satisfaction with nurse’s availability.

| Availability - Nurse | N int./  N cont. | **T3 estimated mean difference int. vs cont. (95% CI)** | **p value** | N int./  N cont. | **T5 estimated mean difference int. vs cont. (95% CI)** |
| --- | --- | --- | --- | --- | --- |
| **Total effects** | 33/30 | 1.6 (-10.1;13.3) | 0.79 | 30/22 | 7.7 (-3.7;19.0) |
| **Sexe**  Male  Female | 7/7  26/23 | 0.0 (-26.3;26.3)  4.4 (-9.7;18.4) | 0.82 | 7/6  23/16 | 14.8 (-13.9;43.6)  6.3 (-6.1; 18.8) |
| **Type of cancer**  Breast  Colorectal  Other | 17/18  11/6  5/6 | 1.3 (-14.2;16.7)  10.0 (-16.7;36.6)  -16.3 (-44.0;11.3) | 0.98 | 18/13  8/7  4/2 | ^1^ |
| **Age**  ≤65  >65 | 21/20  12/10 | 3.3 (-13.1;19.8)  1.0 (-14.6;16.6) | 0.72 | 20/14  10/8 | 13.2 (-1.5;27.9)  -6.9 (-21.5;7.8) |
| **Comorbidity**  None  ≥1 | 12/18  21/12 | -3.8 (-24.6;16.9)  0.7 (-16.4;17.8) | 0.65 | 11/12  19/10 | 1.0 (-20.5;22.6)  15.0 (-0.7;30.8) |

^1^ Data not shown, since the residual variance is zero. Abbreviation: int; intervention group, cont; control group.

**Table C11.** Subgroup effects on patient satisfaction with the relationship with the nurse.

| Relation - Nurse | N int./  N cont. | **T3 estimated mean difference int. vs cont. (95% CI)** | **p value** | N int./  N cont. | **T5 estimated mean difference int. vs cont. (95% CI)** |
| --- | --- | --- | --- | --- | --- |
| **Total effects** | 33/30 | 1.2 (-10.7;13.1) | 0.84 | 30/24 | 1.2 (-9.6;12.1) |
| **Sexe**  Male  Female | 7/7  26/23 | 4.4 (-23.6;32.4)  3.9 (-10.2;18.1) | 0.71 | 7/6  23/18 | 14.8 (-10.4;40.0)  -2.7 (-14.9;9.6) |
| **Type of cancer**  Breast  Colorectal  Other | 17/18  11/6  5/6 | -1.1 (-16.9;14.7)  11.7 (-14.9;38.3)  -17.3 (-42.2;7.5) | 0.91 | 18/14  8/7  4/3 | ^1^ |
| **Age**  ≤65  >65 | 21/20  12/10 | 1.8 (-15.8;19.5)  -0.6 (-16.4;15.1) | 0.83 | 20/16  10/8 | 7.5 (-5.5;20.4)  -14.8 (-33.6;4.0) |
| **Comorbidity**  None  ≥1 | 12/18  21/12 | -1.6 (-21.6;18.3)  1.1 (-17.8;19.9) | 0.75 | 11/14  19/10 | -2.2 (-22.5;18.0)  2.0 (-14.2;18.3) |

^1^ Data not shown, since the residual variance is zero. Abbreviation: int; intervention group, cont; control group.

**Table C12.** Subgroup effects on patient satisfaction with nurse’s attention.

| Attention - Nurse | N int./  N cont. | **T3 estimated mean difference int. vs cont. (95% CI)** | **p value** | N int./  N cont. | **T5 estimated mean difference int. vs cont. (95% CI)** |
| --- | --- | --- | --- | --- | --- |
| **Total effects** | 33/30 | 0.2 (-11.9;12.4) | 0.97 | 30/24 | 9.6 (-0.3;19.4) |
| **Sexe**  Male  Female | 7/7  26/23 | 8.8 (-19.7;37.3)  0.3 (-14.3;14.9) | 0.72 | 7/6  23/18 | 7.8 (-18.6;34.2)  10.9 (0.08;21.7) |
| **Type of cancer**  Breast  Colorectal  Other | 17/18  11/6  5/6 | -3.9 (-19.4;11.7)  18.2 (-7.2;43.6)  -21.9 (-49.3;5.4) | 0.92 | 18/14  8/7  4/3 | 5.4 (-7.2;18.1)  7.5 (-14.4;29.4)  33.3 (11.7;55.0) |
| **Age**  ≤65  >65 | 21/20  12/10 | -1.2 (-18.6;16.3)  -2.3 (-18.8;14.2) | 0.95 | 20/16  10/8 | 11.5 (0.79;22.2)  1.2 (-14.8;17.2) |
| **Comorbidity**  None  ≥1 | 12/18  21/12 | -1.3 (-21.6;18.9)  -0.4 (-19.2;18.5) | 0.71 | 11/14  19/10 | 7.0 (-7.1;21.1)  13.6 (-3.8;31.0) |

Abbreviation: int; intervention group, cont; control group.

**Table C13.** Subgroup effects on patient satisfaction with nurse’s willingness to help.

| Willingness - Nurse | N int./  N cont. | **T3 estimated mean difference int. vs cont. (95% CI)** | **p value** | N int./  N cont. | **T5 estimated mean difference int. vs cont. (95% CI)** |
| --- | --- | --- | --- | --- | --- |
| **Total effects** | 33/30 | 1.4 (-10.7;13.5) | 0.82 | 30/24 | 7.5 (-3.5;18.5) |
| **Sexe**  Male  Female | 7/7  26/23 | 8.8 (-27.4;45.1)  2.0 (-11.7;15.8) | 0.64 | 7/6  23/18 | 7.0 (-23.2;37.3)  8.4 (-3.7;20.5) |
| **Type of cancer**  Breast  Colorectal  Other | 17/18  11/6  5/6 | -4.4 (-18.3;9.4)  18.2 (-11.1;47.4)  -13.3 (-46.8;20.2) | 0.79 | 18/14  8/7  4/3 | 4.0 (-10.4;18.4)  7.1 (-17.9;32.1)  25.0 (-16.9;66.9) |
| **Age**  ≤65  >65 | 21/20  12/10 | 3.3 (-13.9;20.5)  -3.1 (-19.8;13.5) | 0.87 | 20/16  10/8 | 10.0 (-3.1;23.0)  -0.9 (-15.4;13.5) |
| **Comorbidity**  None  ≥1 | 12/18  21/12 | 0.8 (-19.3;20.9)  2.7 (-15.8;21.3) | 0.66 | 11/14  19/10 | 2.2 (-15.7;20.1)  17.4 (-0.3;35.0) |

Abbreviation: int; intervention group, cont; control group.
